# Supplementary material for: Analysis of PPARγ Signaling Activity in Psoriasis
Source: Int J Mol Sci. 2021 Aug 10;22(16):8603. doi: 10.3390/ijms22168603 (PMC8395241; doi:10.3390/ijms22168603)
Supplement: Supplementary file 1 [file ijms-22-08603-s001.zip › Supplemental materials_Analysis of PPARg signaling activity in psoriasis/Pathway models/Models images and html files/PPARG inhibits phenotypes assotiated with psoriasis/142349.html]

T-cell response


# Cell Process T-cell response

|  |  |
| --- | --- |
| URN | urn:agi-cellproc:T-cell%20response |
| Total Entities | 2 |
| Connectivity | 3016 |
| Name | T-cell response |
| ObjectType | Cell Process |

---

|  |  |
| --- | --- |
| ChildConcepts | T-helper cell response |
|  | T-cell proliferative response |

---

|  |  |
| --- | --- |
| Pathway | Complement Alternative Pathway |
|  | Complement Activation by Lectin |
|  | Complement Classical Pathway |
|  | MC1R in anti-Inflammatory Signaling |
|  | Plasmacytoid Dendritic Cell in Diabetes Mellitus Type 1 |
|  | MIR155 Role in Th17-Cell in Multiple Sclerosis |
|  | PPARG inhibits phenotypes assotiated with psoriasis |
|  | efects selected |
|  | Neighbors of T-cell response |
|  | SARS induces Complement Activation |

---

|  |  |
| --- | --- |
| MedScan ID | 4000257 |

---

|  |  |
| --- | --- |
| Alias | T-lymphocyte responses |
|  | T-cell response |
|  | pre-T-lymphocyte responses |
|  | T-lymphoblast response |
|  | T-lymphocyte response |
|  | pre-T-lymphocyte response |
|  | T-lymphoblast responses |
|  | T-cell responses |

---
